# Dietary patterns of adolescents in Germany - Associations with nutrient intake and other health related lifestyle characteristics

**DOI:** 10.1186/1471-2431-12-35

**Published:** 2012-03-22

**Authors:** Almut Richter, Christin Heidemann, Matthias B Schulze, Jutta Roosen, Silke Thiele, Gert BM Mensink

**Affiliations:** 1 Marketing and Consumer Research, Technische Universität München, Alte Akademie 16, 85354 Freising, Germany; 2 Department of Epidemiology and Health Reporting, Robert Koch Institute, Nordufer 20, 13353 Berlin, Germany; 3 Department Molecular Epidemiology, German Institute of Human Nutrition, Arthur-Scheunert-Allee 114-116, 14558 Nuthetal, Germany; 4 Department of Food Economics and Consumption Studies, Christian-Albrechts-Universität Kiel, Olshausenstraße 40, 24098 Kiel, Germany

**Keywords:** Dietary patterns, Adolescents, Nutrition epidemiology, Principal component analysis

## Abstract

**Background:**

The aim of this study was to identify dietary patterns among a representative sample of German adolescents and their associations with energy and nutrient intake, socioeconomic and lifestyle characteristics, and overweight status.

**Methods:**

In the analysis, data from the German Health Interview and Examination Survey for Children and Adolescents were used. The survey included a comprehensive dietary history interview conducted among 1272 adolescents aged 12 to 17 years. Dietary patterns were determined with principal component analysis (PCA) based on 48 food groups, for boys and girls separately.

**Results:**

Three dietary patterns among boys and two among girls were identified. Among boys, high adherence to the 'western' pattern was associated with higher age, lower socioeconomic status (SES), and lower physical activity level (PA). High adherence to the 'healthy' pattern among boys, but not among girls, was associated with higher SES, and higher PA. Among boys, high adherence to the 'traditional' pattern was associated with higher age. Among girls, high adherence to the 'traditional and western' pattern was associated with lower age, lower SES and more hours watching TV per day. The nutrient density of several vitamins and minerals, particularly of B-vitamins and calcium, increased with increasing scores of the 'healthy' pattern among both sexes. Conversely, with increasing scores of the 'western' pattern among boys, most nutrient densities decreased, particularly of fibre, beta-carotene, vitamin D, biotin and calcium. Among girls with higher scores of the 'traditional and western' pattern, nutrient densities of vitamin A, C, E, K and folate decreased. Among boys, high adherence to the 'traditional' pattern was correlated with higher densities of vitamin B_12 _and vitamin D and lower densities of fibre, magnesium and iron. No significant associations between dietary patterns and overweight were found.

**Conclusions:**

Higher scores for dietary patterns characterized by higher consumption of take away food, meat, confectionary and soft drinks ('western' and 'traditional and western') were found particularly among 16- to 17-years old boys and among adolescents with lower SES. These patterns were also associated with higher energy density, higher percent of energy from unsaturated fatty acids and lower percent of energy from carbohydrates as well as lower nutrient densities of several vitamins and minerals. Therefore, nutritional interventions should try to focus more on adolescents with lower SES and boys in general.

## Background

The life stage of childhood and adolescence is important for the establishment of eating behaviours [1-3], which are often carried into adulthood [4-7]. Therefore, diet in the early stage of life influences health not only during the physical development, but also later in life [8-10]. The high prevalence of obesity and nutrition related diseases highlights the need to focus on nutritional interventions early in life [11]. Accordingly, knowledge about actual nutritional intake and diet behaviour among children and adolescents is essential.

Information on dietary patterns reflect the overall nutritional behaviour better than information on single foods or nutrients [12]. Therefore, the analysis of dietary patterns gives a more comprehensive impression of the food consumption habits within a population. Dietary guidelines based on preferred dietary patterns may be easier to understand and transported to the public than nutrient intake recommendations, because people are aware of their food consumption but not their nutrient intake.

Adolescence is a special stage of life often full of personal changes. Thus, adolescents could benefit from recommendations which are close to the existing food habits of their age group.

Associations between nutrition and health are complex and often influenced by many factors. Often there is a high correlation between nutrients and between foods which may complicate the interpretation of all relevant intake variables within multiple regression analysis. The application of statistical techniques to reduce the complexity of diet to a smaller number of dimensions, such as principal component analysis, can be useful in such cases. Resulting dietary patterns can than be used to evaluate associations between nutrition and health related measures (e. g. anthropometric measures) and diseases.

Most of the previous studies on dietary patterns of adolescents are based on relatively small or non-representative samples, e. g. school classes [4,13-21]. Until now, there have been only a few European studies on population based samples, including studies from North Ireland among persons older than 16 years [22] and studies of children and adolescents from Spain [23], the Balearic Islands [24] and Scotland [25]. Outside Europe, dietary patterns in representative samples of adolescents were analysed in Mexico [26], Australia [27] and Korea [28,29].

Therefore, the aim of this study was to identify dietary patterns among a representative sample of German adolescents and their associations with energy and nutrient intake, socioeconomic and lifestyle characteristics, and overweight status.

## Methods

### Study population

The German Health Interview and Examination Survey for Children and Adolescents (KiGGS) is a population-based, nationally representative, cross-sectional study [30]. The sample was drawn with a two-stage clustered and stratified sampling procedure. In the first stage, 167 sample points (initially 150 and later 17 additional points) representative for German communities were selected and stratified by federal state and community size. In the second stage, for every age, almost the same number of participants was randomly selected from the population registries. From May 2003 to May 2006, a total of 17,641 children and adolescents aged 0 to 17 years, participated in KiGGS. The overall response rate was 66.6%. The survey was approved by the Federal Office for Data Protection and by the ethics committee of Charitè University Medicine. Each parent and participant gave informed written consent before enrolment into the survey. Design, methods and response analyses are described in detail elsewhere [31].

For the present analysis, we included a subsample of the KiGGS participants (Eating Study as KiGGS Module, EsKiMo). The participants of EsKiMo were randomly selected from the KiGGS participants in the original 150 sample points, stratified by age and sample point. A net sample size of 100 participants per age and sex was intended. In total, information of 1234 children aged 6 to 11 years and 1272 adolescents aged 12 to 17 years was obtained. EsKiMo was conducted from January to December 2006 by the Robert Koch Institute and the University of Paderborn [32]. As children and adolescents differ in their ability and willingness to cooperate and in their personal circumstances (e. g. frequency of meals outside home), two different tools for data collection were used. Parents of participants younger than 12 years were asked to complete dietary records on three given consecutive days. Participants 12 years of age and older took part in a comprehensive dietary assessment. The present analyses are based on the 622 boys and 650 girls aged ≥ 12 to < 18 years who took part in EsKiMo. The participation rate of EsKiMo for this age group was 64.7%.

### Data collection

KiGGS includes a range of physical examinations and tests as well as questionnaires filled out by parents and children aged 11 years and older. Parents were asked about their educational and occupational status and available household family income. Socioeconomic indicators were computed from each of these three components ranging from 1 to 7. A summary family socioeconomic status (SES) index was calculated for every participant ranging from a minimum of 3 to a maximum of 21. A higher SES index corresponds to a higher status [33]. City size was assessed and grouped into small (under 5,000 inhabitants), small and middle-sized (5,000 to 99,999 inhabitants) and big cities (100,000 inhabitants or more).

Participants of EsKiMo 12 years of age and older, were interviewed by trained nutritionists using the Dietary Interview Software for Health Examination Studies (DISHES), a computerised face-to-face modified dietary history instrument designed to assess usual dietary intake within a reference period of the last four weeks. DISHES was developed at the Robert Koch Institute and was used in several nutrition surveys [34,35]. The relative validity of DISHES for adults was assessed in comparison with 3-d weighted dietary records and a 24-h dietary recall and revealed correlations for nutrient intakes in a reasonable range (0.34 to 0.69 for 3-d weighted dietary records, 0.27 to 0.65 for 24-h recall) [34]. In the DISHES-interviews, firstly, the usual meal patterns of the participant were assessed. Secondly, food groups consumed during each meal were obtained by a check list. Lastly, following the meal structure of the day, the frequencies and amounts of all specific foods and drinks consumed during each meal were assessed. Estimations of portion sizes are facilitated through use of standardized tableware models and a picture book with different reference portion sizes. In DISHES, all food items are coded automatically with the German Food Code and Nutrient Database (BLS II.3) [36]. DISHES was updated and adapted for this study and the target group of adolescents. For instance, additionally foods, not included in the BLS but consumed by adolescents, were incorporated. The nutrition interviews were conducted primarily at the adolescent's home or in cases when this was not possible, in a survey van. For this, every sample point was visited by one of the interviewers. The interviews were equally distributed over the year, thus, the issue of seasonality was accounted for on the group level. The average duration of the interview was 49 minutes. Participants received 10 € as an incentive along with a personal evaluation of their interview (e. g. nutrient intakes in comparison to reference values). To improve data quality, voice recorders were used to check participant answers if questionable data were detected.

In addition to the DISHES interview, EsKiMo included questions about specific nutritional, lifestyle and behavioural aspects. These questions referred to dietary supplement use ('Do you use dietary supplements (vitamins, minerals) e. g. pills, drops? Which products? How often did you take these products during the last four weeks?'), frequency of family meals ('Do you have joined meals at home? Which meals: breakfast, lunch, dinner? How often? Every day, three to five times a week, one to two times a week, less, never'), frequency of eating a warm lunch at school ('Do you have the possibility to eat a warm lunch at school? If yes: How often do you eat a warm lunch at school? Daily, tree to four times a week, one to two times a week or more seldom') and self-reported cooking skills ('How good are your cooking skills? Very good, good, ordinary, not good, bad, I don't cook'). Questions about leisure time activities referred to frequency of physical activity and time spent watching television per day ('In your leisure time, how often are you physically active in such a way that you start to sweat or become slightly out of breath? Never, almost every day, about three to five times a week, about one to two times a week, about one to two times a month' and 'How long do you usually watch TV per day? Never, 30 minutes, one to two hours, three to four hours, more'). Body mass index (BMI) was calculated from self-reported body height and weight. Participants with a BMI above the 90^th ^percentile of the age and gender specific reference values [37] were categorised as overweight.

### Statistical analyses

In the DISHES interviews, 2280 different foods and beverages were reported. These foods were combined into 48 food groups (Table 1). Therefore, commonly used food groups were differentiated according to similarities in nutrient profiles. For every food group, the mean amount consumed daily in grams was calculated for each individual and standardized to a mean of 0 and standard derivation of 1.

**Table 1:** Factor loadings* of the food groups in the dietary patterns (principal components) identified among German adolescents

	Dietary patterns				
	Boys (n = 622)			Girls (n = 650)	
Food groups	western	healthy	traditional	healthy	Traditional and western
Pizza^†^	0.66				0.32
Doner kebab	0.59				0.26
Burger	0.57				0.29
Soft drinks	0.55	-0.24	0.29		0.46
French fries	0.53				0.32
Alcoholic drinks	0.42		0.25		
Chicken	0.41	0.32		0.51	
Ketchup^‡^	0.40				0.26
Salty snacks	0.37				
Meat (except chicken)	0.33	0.28	0.37	0.21	0.46
Pasta	0.33			0.26	0.45
Confectionary	0.33				0.46
Sausages^§^	0.27				0.46
Legumes	0.25	0.41		0.47	0.23
Warm sauces	0.25		0.21	0.31	0.37
Rice	0.23	0.59		0.65	
Wholemeal bread	-0.29				-0.21
Salad vegetables		0.59		0.44	
Fruits		0.57		0.35	
Other vegetables^||^		0.53	0.27	0.43	
Vegetable oil		0.52		0.61	
Mushrooms		0.37		0.35	
Soup		0.35		0.59	0.28
Grain products^¶^		0.35			
Fish		0.28	0.31	0.38	
Cake/cookies		0.25	0.23		0.35
Eggs		0.25	0.35	0.33	0.21
Potatoes		0.25	0.56	0.29	0.42
Nuts		0.25			
Falafel**		0.24		0.24	
Water		0.23		0.31	-0.36
Processed meat			0.69		0.47
White bread^††^			0.51		0.38
Margarine			0.38		
Cheese			0.33		
Butter			0.28		
Meat salad^‡‡^			0.27		
Dessert, Ice-Cream			0.25		0.25
Yoghurt			0.25		
Coffee			0.22		
Breakfast cereals					
Jam					0.24
Juices					
Milk					
Other milk products^§§^					0.20
Pancakes					
Tea				0.22	
Vegetarian dishes^|| ||^				0.36	

**Variance explained (%)**	8.2	5.1	4.8	7.6	5.6

* Factor loadings with absolute values < 0.2 are not shown for simplicity, absolute values > 0.4 are underlined

† Pizza, onion tart

‡ Ketchup, mayonnaise, mustard, other cold sauces

§ Hot dog, grilled fried sausage, curried sausage, meatballs

|| Tomato, cucumber, pepper, asparagus, garlic, avocado, carrot, cabbage, mixed pickles, olives

¶ Bulgur, popcorn, rice waffle

** Falafel, vegetarian doner, Turkish pizza

†† Wheat bread, mixed bread, bread rolls

‡‡ Salad with meat, chicken, eggs or fish

§§ Cream cheese, curd cheese, buttermilk, kefir, soured milk, cream, concentrated milk

|| ||Soya, tofu, vegetarian spread

From previous analyses it was known that food consumption was different between sexes in this age group [38]. Therefore, the analyses were separately conducted for boys and girls. Dietary patterns were identified using principal component analysis (PROC FACTOR method = prin) on the 48 food groups. The resulting components were linear combinations of the included variables and explained as much of the variation in the original variables as possible. The components were rotated by an orthogonal transformation (resulting in uncorrelated components) to achieve a simpler structure with greater interpretability. To identify the number of principal components to be retained, the following criteria were used: the criterion of eigenvalues exceeding 1 (the interpretation of this criterion being that each component should explain a larger amount of variance than a single standardized variable in order to be retained), the scree plot (which is a graphical presentation of eigenvalues) and the interpretability of each component [39]. For good interpretability of each component, an adequate number of food groups with high loadings within a component are necessary. According to Hatcher 2007 [39], components with at least 3 relevant loadings, which are loadings greater than or equal to |0.4|, were selected. The dietary pattern score is based on the sum of the individual, standardized intake of each food group weighted by the loading of the food group. The scores rank individuals according to the degree to which they conformed to each dietary pattern. The scores were categorized into quartiles and labelled according to the food groups with high loadings. Each participant had a score for all identified dietary patterns.

To estimate nutrient and energy intake by quartiles of dietary patterns, parameters were calculated using data from the German Food Code and Nutrient Data Base (BLS II.3) [36]. To determine the nutrient content of foods eaten by the study participants which were not incorporated in the BLS, an additionally database was developed using different sources e. g. product information of the food producer.

Dietary patterns were derived without considering dietary supplements as a food group. Therefore, the analyses on nutrient intakes were also performed without taking the contribution of supplements into account.

The internal validity of the pattern structure was tested by calculating Cronbach's coefficient alpha [39].

To correct for non-response and disproportionate sample drawing, a specific weighting factor was used for all analyses [31]. Since the sample was based on a clustered and stratified design, all analyses were performed with complex survey procedures in order to take the sampling design into account.

P values for trend across quartiles of dietary pattern scores were calculated in linear regression models using the survey procedure (PROC SURVEYREG). Associations between categorical variables were tested by calculation of the X^2 ^test. P values < 0.05 were considered statistically significant. For all statistical analyses the SAS System for Windows 9.2 (SAS Institute, Cary, NC, USA) was used.

## Results

### Dietary patterns

We identified three major components (patterns) for boys and two for girls. These components accounted for 18.1% of the variance in food group intake in boys and 13.2% in girls. The food group loadings for each component are presented in Table 1.

Among boys, the first component was positively correlated with the intake of take away foods (pizza, doner kebab, burgers, French fries), ketchup, chicken, and other meat, pasta, alcoholic and soft drinks, salty snacks and confectionery items. This pattern was labelled 'western'. The second component was named 'healthy' as relatively high positive loadings were observed for intakes of fruits, vegetables, legumes, mushrooms, chicken, rice, vegetable oil, soup, and grain products. The third component in boys could be described as a traditional German diet, reflecting a pattern of eating traditional warm dishes and sandwiches. For this 'traditional' pattern, we obtained relatively high positive loadings for processed meat, potatoes, white bread, margarine, meat (except chicken), eggs, cheese, and fish.

Among girls, the first component was positively correlated with the intake of rice, vegetable oil, soup, chicken, legumes, vegetables, fruits, and mushrooms similar to the 'healthy' pattern of boys. Among girls, this 'healthy' pattern was also positively correlated with vegetarian dishes, eggs, fish, water and warm sauces. The second component among girls, called ‚traditional and western' pattern, was positively correlated with potatoes, warm sauces, meat (except chicken), white bread, processed meat, as well as pizza, French fries, sausages, soft drinks, confectionary, cake/cookies and negatively correlated with water.

Cronbach's alpha indicated that there was moderate inter-item reliability (among boys: 0.71 for the 'western' pattern, 0.62 for the 'healthy' pattern, 0.61 for the 'traditional' pattern; among girls: 0.67 for the 'healthy' pattern, 0.61 for the 'traditional and western' pattern).

### Characteristics

Sample characteristics of the adolescents according to quartiles of each dietary pattern score are presented in Table 2 for boys and Table 3 for girls.

**Table 2:** Socio-demographic and behavioural sample characteristics of German boys according to quartiles of dietary pattern scores

Dietary patterns		Western					Healthy					Traditional				
		**Q1**	**Q2**	**Q3**	**Q4**	**p**	**Q1**	**Q2**	**Q3**	**Q4**	**p**	**Q1**	**Q2**	**Q3**	**Q4**	**p**

**n**		155	156	156	155		155	156	156	155		155	156	156	155	
**Age (mean)^†^**		14.0	14.0	14.5	15.5	**<.0001**	15.0	14.3	14.5	14.5	0.1218	14.1	14.1	15.0	15.2	**<.0001**
**Community size (number of subjects, %)^‡^**																
< 5000 inhabitants		28.0	30.8	18.1	23.1		29.7	33.1	21.1	16.2		17.9	23.8	25.4	32.9	
5000 - < 100000 inhabitants		18.9	23.9	25.9	31.3		27.3	25.5	24.0	23.2		25.1	28.0	25.5	21.4	
> 100000 inhabitants		24.2	22.1	25.2	28.6	0.1931	20.1	19.9	26.8	33.2	**0.033**	38.2	20.7	23.6	17.6	**0.0079**
**Socioeconomic status (3-21) ^§†^**		12.7	11.5	12.4	11.4	**0.0341**	11.2	12.0	12.4	12.4	**0.0226**	12.1	12.4	12.1	11.2	0.0988
**Education (number of subjects %)^‡^**																
Grammar school		33.5	21.7	25.9	18.9		12.1	24.0	33.7	30.2		30.2	28.9	22.1	18.7	
Secondary school**^||^**		15.7	32.0	22.3	30.0		33.1	26.2	18.4	22.3		30.3	23.4	21.5	24.8	
Others		21.4	22.3	30.8	25.5	**0.0008**	28.1	27.0	16.1	28.7	**0.0001**	22.6	28.6	30.1	18.7	0.4305
**Dietary supplement user (number of subjects, %)^‡¶^**		16.4	23.4	26.7	33.6	0.4234	17.1	19.7	26.6	36.6	**0.0109**	24.7	23.8	24.5	27.0	0.6456
**Joint family meals (number of subjects, %)^‡^**																
breakfast	at least three or five times a week	29.2	30.8	21.2	18.8		23.2	27.4	23.5	26.0		23.3	24.2	24.4	28.2	
	more seldom or never	19.0	21.2	25.8	33.9	**0.0004**	26.1	24.4	24.8	24.7	0.8159	29.8	25.6	25.7	18.9	0.1033
lunch	at least three or five times a week	24.9	27.1	23.8	24.2		23.7	26.1	23.7	26.4		28.4	23.1	26.2	22.3	
	more seldom or never	19.6	21.4	24.7	34.4	0.0678	27.0	24.5	25.0	23.5	0.7651	26.9	27.4	24.1	21.6	0.7472
dinner	at least three or five times a week	24.3	25.3	24.0	26.5		19.9	25.0	25.8	29.3		28.5	25.2	22.8	23.5	
	more seldom or never	18.2	22.8	24.9	34.0	0.2888	38.4	26.4	20.4	14.8	**<.0001**	26.2	24.8	30.5	18.5	**<.0001**
**Lunch at school (number of subjects, %)^‡^**																
	Five times a week	7.8	7.1	10.6	6.8		7.3	8.0	7.4	9.7		1.9	13.3	7.2	9.0	
	at least one or two times a week	24.8	24.5	18.6	36.2		23.1	23.3	21.5	35.3		26.0	24.7	26.3	26.8	
	more seldom or never	67.5	68.4	70.8	57.1	0.3754	69.6	68.7	71.1	55.0	0.4794	72.1	62.0	66.5	64.2	0.2698
**Cooking skills (number of subjects, %)^‡^**																
	very good	23.6	17.4	27.0	32.0		28.6	27.2	21.5	22.7		24.0	20.1	32.2	23.7	
	good	21.6	26.8	22.9	28.7		24.6	21.9	26.5	27.0		27.7	25.0	20.1	27.2	
	worse	21.4	28.5	23.8	26.3	0.4909	24.1	30.4	22.9	22.6	0.2706	31.7	31.4	27.1	9.8	0.1468
**Physical activity (hours per week)^†^**	6.0	5.8	4.2	5.2	**0.08**	5.0	4.8	5.5	6.0	**0.0398**	4.7	5.6	4.7	6.4	**0.042**	
**Time spent watching TV a day^‡^**																
	0 to 30 minutes	25.7	26.1	22.6	25.7		24.9	25.3	20.1	29.8		30.3	29.3	23.3	17.1	
	1 to 2 hour	24.8	23.4	24.3	27.6		23.0	26.9	26.3	23.8		28.0	23.8	25.9	22.3	
	3 to 4 hour or more	13.2	26.5	25.3	35.1	0.2590	31.9	21.7	23.4	23.1	0.4584	24.4	24.7	24.1	26.8	0.7075
**Overweight adolescents (number of subjects, %)^‡^**	21.3	22.5	29.9	26.3	0.7168	28.0	22.9	17.3	31.7	0.3934	36.6	19.6	20.4	23.4	0.2774	

^† ^test for trends

^‡ ^Χ² statistics

^§ ^Index according to Winkler [33]

^║ ^Haupt- oder Realschule

^¶ ^at least one time supplement use within a reference period of 4 weeks

Boys in the higher quartiles of the 'western' pattern score were older and attended grammar school less often than those in the lower quartiles. The families of these boys had a lower SES compared to boys in the lower quartiles of this pattern. With increasing scores of the 'western' pattern, boys were less physically active and had family breakfast less often three to five times a week (Table 2).

Boys in the higher quartiles of the 'healthy' pattern more often resided in communities with more than 100,000 inhabitants, more often attended grammar school and more often live in families with a higher SES than boys in the lower quartiles of this pattern score. Additionally, these boys were more physically active, took more often dietary supplements and more often had family dinners three to five times a week than boys in the lower quartiles of this pattern.

Boys in the higher quartiles of the 'traditional' pattern were older, more often resided in communities with less than 5,000 inhabitants and less often had family dinners three to five times a week than boys in the lower quartiles of this pattern score.

Among girls, no significant differences between those with lower and higher dietary pattern scores of the healthy pattern were found (Table 3).

**Table 3:** Socio-demographic and behavioural sample characteristics of German girls according to quartiles of dietary pattern scores

Dietary patterns		Healthy					Traditional and Western				
		**Q1**	**Q2**	**Q3**	**Q4**	**p**	**Q1**	**Q2**	**Q3**	**Q4**	**p**

**n**		162	163	163	162		162	163	163	162	
**Age (mean) ^†^**		14.6	14.3	14.6	14.8	0.2309	15.0	14.4	14.6	14.3	**0.0108**
**Community size (number of subjects, %)^‡^**											
< 5000 inhabitants		29.4	25.9	29.8	14.9		29.6	20.3	22.9	27.2	
5000 - < 100000 inhabitants		25.1	26.3	20.6	27.9		24.5	26.1	25.8	23.7	
> 100000 inhabitants		22.0	21.7	22.4	33.9	0.0582	28.4	25.9	23.4	22.3	0.8168
**Socioeconomic status (3-21) ^§†^**		11.5	11.0	12.8	11.4	0.4352	12.8	11.7	12.2	9.8	**<.0001**
**Education (number of subjects %)^‡^**											
Grammar school		23.6	20.2	27.5	28.8		31.9	23.4	27.7	17.0	
Secondary school^**||**^		27.0	30.5	19.5	23.0		22.1	23.0	21.1	33.8	
Others		28.9	24.4	17.4	29.3	0.1290	24.5	31.5	27.2	16.8	**0.0019**
**Dietary supplement user (number of subjects, %)^‡¶^**		19.3	22.4	23.2	35.1	0.1759	29.9	18.1	22.6	29.5	0.2568
**Joint family meals (number of subjects, %)^‡^**											
breakfast	at least three or five times a week	23.7	25.5	27.4	23.5		27.7	23.7	24.1	24.6	
	more seldom or never	25.9	25.1	19.9	29.1	0.2244	25.5	25.7	24.9	24.0	0.9405
lunch	at least three or five times a week	23.9	26.9	21.6	27.6		26.3	26.2	23.2	24.3	
	more seldom or never	26.6	23.3	23.4	26.8	0.7373	26.1	23.5	26.4	24.1	0.8447
dinner	at least three or five times a week	24.5	26.8	21.7	27.1		26.4	24.1	25.9	23.6	
	more seldom or never	26.2	21.6	24.7	27.6	0.6814	26.4	26.6	21.4	25.6	0.7490
**Lunch at school (number of subjects, %)^‡^**											
	Five times a week	5.0	6.6	6.1	3.5		2.0	6.7	5.0	7.9	
	at least one or two times a week	14.0	27.4	16.8	9.1		17.2	19.3	13.7	14.6	
	more seldom or never	81.1	66.0	77.1	87.4	0.0531	80.8	74.0	81.3	77.5	0.5885
**Cooking skills (number of subjects, %)^‡^**											
	very good	22.8	24.7	19.8	32.8		29.8	21.6	22.6	26.1	
	good	26.3	24.1	25.2	24.4		24.2	26.1	26.1	23.6	
	worse	28.4	29.8	20.0	21.8	0.1413	26.4	28.2	24.5	20.9	0.3977
**Physical activity (hours per week)^†^**		3.5	4.2	3.6	3.2	0.2021	4.3	3.3	3.4	3.5	0.1801
**Time spent watching TV a day^‡^**											
	0 to 30 minutes	20.9	22.5	27.2	29.3		35.1	25.3	20.1	19.6	
	1 to 2 hour	24.4	27.1	22.4	26.2		27.0	25.3	26.1	21.6	
	3 to 4 hour or more	32.7	21.1	19.0	27.2	0.4633	16.0	22.4	24.9	36.7	**0.0144**
**Overweight adolescents (number of subjects, %)^‡^**		24.1	31.8	26.0	18.1	0.3941	31.5	25.6	22.8	20.1	0.7781

^† ^test for trends

^‡ ^Χ² statistics

^§^ Index according to Winkler [33]

^║ ^Haupt- oder Realschule

^¶ ^at least one time supplement use within a reference period of 4 weeks

With higher scores of the 'traditional and western' pattern, girls were younger, had a lower SES and less often attended grammar school. In addition, girls in the higher quartiles of the 'traditional and western' pattern more often watched three to four or more hours television per day than those in the lower quartiles of this pattern.

No associations between dietary pattern scores and frequency of lunch at school, cooking skills, overweight prevalence, or the season of the nutrition interview (data not shown) were observed.

### Energy and nutrient intake

Mean daily energy and nutrient intakes according to quartiles of dietary pattern scores are presented in Table 4 for boys and Table 5 for girls. Fat, protein, carbohydrates and their subgroups are presented as percentages of energy intake whereas energy, fibre, vitamins and minerals are given as nutrient densities.

**Table 4:** Mean daily energy and nutrient density* according to quartiles of the dietary pattern scores identified among German boys

	Western pattern					Healthy pattern					Traditional pattern				
	Q1	Q2	Q3	Q4	P for trend	Q1	Q2	Q3	Q4	P for trend	Q1	Q2	Q3	Q4	P for trend
**Energy intake (MJ)**	11.0	11.2	12.1	16.5	**<.0001**	12.7	11.4	12.7	14.9	**<.0001**	10.2	11.3	14	16.8	**<.0001**
**% of energy**															
Total fat	32.5	34.3	34.1	33.8	0.283	33.6	34.3	33.1	33.8	0.955	31.8	32.9	34.7	35.7	**<.0001**
Saturated fatty acids (SFA)	14.0	15.0	14.5	13.8	0.1016	14.4	14.9	14.1	14.0	0.1629	13.3	14.0	14.9	15.4	**<.0001**
Unsaturated fatty acids	11.2	11.9	12.1	12.1	**0.007**	11.9	12.1	11.6	11.8	0.5917	11.1	11.7	12.1	12.7	**<.0001**
Polyunsaturated fatty acids (PUFA)	4.7	4.8	4.9	5.0	0.1016	4.6	4.7	4.8	5.3	**0.001**	4.9	4.7	4.9	5.0	0.5486
Protein	13.7	13.4	13.9	13.8	0.360	13.0	13.6	13.9	14.3	**<.0001**	13.7	13.6	13.7	13.9	0.511
Total Carbohydrates	52.5	50.9	50.4	49.4	**0.0003**	50.9	50.4	51.2	50.4	0.699	53.0	52.0	49.2	48.2	**<.0001**
Monosaccharide	11.3	11.1	11.6	11.2	0.9334	11.9	10.9	11.2	11.1	0.4272	11.8	11.8	10.9	10.5	0.0213
Disaccharide	14.7	16.0	15.2	15.1	0.9302	16.2	15.3	15.3	14.3	**0.0038**	15.6	15.8	15.1	14.5	0.068
Polysaccharide (absorbable)	26.1	23.7	23.5	23.3	**0.0016**	23.1	24.1	24.5	24.5	0.0785	25.7	24.1	23.1	23.0	**0.0004**
Alcohol	0.5	0.4	0.8	1.9	**<.0001**	1.6	0.8	0.9	0.6	**0.001**	0.5	0.6	1.5	1.4	**0.001**
**Energy and nutrient density**															
Energy (kJ/100 g)^†^	604.4	656.6	711.7	725.3	**<.0001**	762.4	692.7	656.1	598.7	**<.0001**	657.1	679	675.1	707.5	**0.001**
Fibre (not absorbable) (g/MJ)	3.0	2.3	2.2	1.9	**<.0001**	1.8	2.2	2.5	2.7	**<.0001**	2.4	2.4	2.2	2.2	**0.021**
Vitamin A (μg/MJ)	144.4	133.7	120.5	113.4	**<.0001**	106.6	118.0	136.0	148.4	**<.0001**	119.0	127.1	128.8	134.7	0.052
Beta-Carotene (μg/MJ)	471.6	403.9	373.9	320.9	**<.0001**	241.1	332.7	450.0	533.4	**<.0001**	408.0	397.9	368.9	371.5	0.268
Vitamin C (mg/MJ)	18.9	16.7	14.0	14.4	**0.009**	12.0	15.5	16.7	19.5	**<.0001**	15.8	15.8	16.3	15.4	0.875
Vitamin D (μg/MJ)	0.3	0.2	0.2	0.2	**0.006**	0.2	0.2	0.2	0.3	**0.015**	0.2	0.2	0.2	0.3	**0.001**
Vitamin E (μg/MJ)	1637.4	1521.5	1428.3	1296.6	**<.0001**	1271.4	1392.3	1559.8	1623.0	**<.0001**	1550.4	1471.6	1386.9	1413.7	0.077
Vitamin K (μg/MJ)	34.3	29.3	28.6	27.1	**0.0001**	21.6	27.8	31.2	38.1	**<.0001**	30.3	29.4	29.8	28.6	0.297
Vitamin B_1 _(μg/MJ)	208.3	187.4	179.0	160.6	**<.0001**	166.4	181.4	194.1	187.7	**0.042**	180.3	184.7	175.1	189.7	0.496
Vitamin B_2 _(μg/MJ)	242.0	217.4	190.7	180.6	**<.0001**	193.7	203.2	215.5	211.0	0.124	210.4	202.4	200.9	208.8	0.888
Niacin (μg/MJ)^‡^	3464.1	3285.9	3300.0	3242.6	0.184	3052.5	3200.5	3528.8	3497.9	**0.0003**	3349.2	3288	3240	3391.8	0.808
Vitamin B_5 _(μg/MJ)	786.2	701.2	644.1	594.2	**<.0001**	593.0	654.5	733.6	723.6	**0.001**	694.4	681.1	641.8	681.6	0.600
Vitamin B_6 _(μg/MJ)	267.3	245.0	227.5	218.7	**0.006**	214.5	222.8	262.5	253.9	**0.002**	243.0	237.7	226.6	245.2	0.978
Biotin (μg/MJ)	9.3	8.0	7.5	6.3	**0.002**	6.3	7.6	8.7	8.1	**0.040**	8.3	8.0	6.9	7.5	0.214
Folate (μg/MJ)	35.9	29.8	27.5	26.8	**<.0001**	26.5	27.5	31.7	33.3	**0.0002**	31.2	29.1	28.8	29.5	0.431
Vitamin B_12 _(μg/MJ)	0.6	0.6	0.5	0.5	0.419	0.5	0.5	0.6	0.5	0.999	0.5	0.5	0.6	0.6	**<.0001**
Calcium (mg/MJ)	141.6	128.2	112.4	101.4	**<.0001**	108.5	123.9	126.2	120.0	0.069	125	117.9	118.6	115.6	0.103
Magnesium (mg/MJ)	50.4	42.8	41.3	37.2	**<.0001**	36.0	43.0	45.5	45.8	**<.0001**	45.4	43.0	41.0	40.1	**0.0002**
Iron (μg/MJ)	1646.2	1419.0	1427.5	1386.5	**<.0001**	1333.2	1412.9	1521.3	1586.1	**<.0001**	1511.3	1476.0	1425.0	1426.1	**0.040**

*Does not include nutrient intake from dietary supplements

^† ^Foods without beverages

^‡ ^Niacin equivalent

**Table 5:** Mean daily energy and nutrient density* according to quartiles of the dietary pattern scores identified among German girls

	Healthy pattern					Traditional and western pattern				
	**Q1**	**Q2**	**Q3**	**Q4**	**P for trend**	**Q1**	**Q2**	**Q3**	**Q4**	**P for trend**
**Energy intake (MJ)**	9.2	8.9	9.6	11.0	**<.0001**	7.3	8.5	10.0	13.3	**<.0001**
**% of energy**										
Total fat	32.4	32.3	31.8	32.2	0.864	30.2	31.2	32.9	34.6	**<.0001**
Saturated fatty acids (SFA)	14.2	14.0	13.8	13.3	**0.031**	12.6	13.6	14.3	14.8	**<.0001**
Unsaturated fatty acids	11.4	11.1	10.8	10.7	**0.0309**	10.0	10.6	11.3	12.2	**<.0001**
Polyunsaturated fatty acids (PUFA)	4.4	4.7	4.8	5.7	**0.0026**	5.3	4.6	4.8	4.9	0.5288
Protein	12.4	13.0	13.0	13.6	**0.001**	13.3	12.8	12.8	13.1	0.593
Total Carbohydrates	53.4	53.3	53.4	52.5	0.319	54.6	54.2	52.6	50.8	**<.0001**
Monosaccharide	13.4	12.1	12.2	12.5	0.4261	13.7	12.6	12.0	11.9	**0.0251**
Disaccharide	16.6	17.2	15.6	15.8	0.0674	15.1	17.2	16.5	16.5	0.1092
Polysaccharide (absorbable)	23.5	23.9	25.2	24.0	0.4346	25.1	24.4	24.0	22.9	**0.0126**
Alcohol	0.9	0.5	0.8	0.6	0.293	0.9	0.7	0.7	0.5	**0.011**
**Energy and nutrient density**										
Energy (kJ/100 g)^†^	695.2	630.3	589.8	540.2	**<.0001**	547.3	601.9	635.4	673.8	**<.0001**
Fibre (not absorbable) (g/MJ)	2.2	2.6	3.0	3.1	**<.0001**	3.4	2.7	2.4	2.2	**<.0001**
Vitamin A (μg/MJ)	138.7	147.7	165.9	180.8	**<.0001**	191.0	148.6	146.2	145.8	**0.0003**
Beta-Carotene (μg/MJ)	402.9	500.6	603.4	702.5	**<.0001**	747.3	532.3	461.8	459.0	**<.0001**
Vitamin C (mg/MJ)	18.0	20.1	22.5	23.9	**0.0002**	26.4	21.7	17.6	18.4	**<.0001**
Vitamin D (μg/MJ)	0.2	0.2	0.2	0.3	**0.001**	0.2	0.2	0.2	0.2	0.735
Vitamin E (μg/MJ)	1573.8	1683.0	1674.1	1893.1	0.078	2078.0	1618.9	1543.4	1573.0	**0.008**
Vitamin K (μg/MJ)	25.3	31.2	38.8	46.0	**<.0001**	41.7	35.7	32.6	31.3	**<.0001**
Vitamin B_1 _(μg/MJ)	191.5	193.8	179.0	176.3	0.229	206.3	176.0	171.8	185.0	0.386
Vitamin B_2 _(μg/MJ)	225.3	232.0	212.9	209.0	0.222	246.0	211.5	208.9	210.7	0.203
Niacin (μg/MJ)^‡^	3200.0	3373.0	3249.8	3366.4	0.547	3653.4	3142.5	3075.1	3305.5	0.280
Vitamin B_5 _(μg/MJ)	728.6	758.2	727.6	731.8	0.905	855.9	705.1	673.1	703.8	0.129
Vitamin B_6 _(μg/MJ)	244.6	254.5	247.4	248.8	0.956	288.1	236.8	225.3	242.6	0.177
Biotin (μg/MJ)	9.1	9.4	8.3	7.8	0.251	10.7	7.9	7.6	8.3	0.284
Folate (μg/MJ)	31.3	34.1	34.3	36.6	**0.040**	40.6	33.3	31.3	30.8	**0.0028**
Vitamin B_12 _(μg/MJ)	0.5	0.5	0.5	0.5	**0.002**	0.5	0.5	0.5	0.5	0.275
Calcium (mg/MJ)	123.2	136.7	145.0	141.4	**0.006**	173.0	133.3	130.5	105.7	**<.0001**
Magnesium (mg/MJ)	41.4	46.4	50.9	52.0	**<.0001**	60.9	46.5	44.3	37.7	**<.0001**
Iron (μg/MJ)	1427.2	1518.7	1584.1	1675.0	**<.0001**	1713.9	1560.2	1482.6	1440.4	**<.0001**

*Does not include nutrient intake from dietary supplements

^† ^Foods without beverages

^‡ ^Niacin equivalent

With increasing scores of the 'western' pattern among boys intake of carbohydrates and polysaccharides decreased, whereas energy density, unsaturated fatty acids and alcohol increased. For this pattern, the micronutrient densities decreased from the lowest to the highest quartile, particularly of fibre, beta-carotene, vitamin D, biotin and calcium.

With increasing scores of the 'healthy' pattern among boys, energy density and intake of alcohol decreased and intake of polyunsaturated fatty acids (PUFAs), intake of proteins and nutrient density (except for vitamin B_2_, vitamin B_12 _and calcium) increased.

Among boys, increasing scores of the 'traditional' pattern were associated with a higher energy density. Intake of total fat, saturated and unsaturated fatty acid, alcohol as well as vitamin B_12 _and vitamin D increased with increasing scores, whereas intake of total carbohydrates, intake of monosaccharides, polysaccharides, fibre, magnesium and iron decreased with increasing scores.

As for the 'healthy' pattern among boys, energy density decreased and protein density increased with increasing scores of the 'healthy' pattern among girls. In addition, increasing scores of the 'healthy' pattern among girls were associated with a lower intake of saturated and unsaturated fatty acids and a higher intake of PUFAs. Micronutrient densities increased from the lowest to the highest quartile of the 'healthy' pattern except for vitamin E and most of the B-vitamins. The nutrient density of vitamin B_12 _was significant lower with increasing patterns scores.

With increasing scores of the 'traditional and western' pattern among girls, energy density and intake of total fat, saturated and unsaturated fatty acids increased, whereas intake of vitamin A, C, E, K and folate as well as intake of alcohol and intake of total carbohydrates, monosaccharides and polysaccharides decreased.

## Discussion

With principal component analyses, we identified three dietary patterns among boys and two among girls in a population-based sample of German adolescents. Dietary patterns showed significant associations with nutrient intake. Because of the higher densities of vitamins, minerals and fibre, the 'healthy' patterns are more favourable compared to the 'western' and 'traditional and western' patterns which were associated with higher energy density, higher percent of energy from unsaturated fatty acids, lower percent of energy from carbohydrates and lower nutrient densities of several vitamins and minerals. The 'traditional' pattern was characterised by favourable as well as less favourable aspects.

Most of the dietary patterns were associated with health related lifestyle characteristics e. g. physical activity and frequency of family meals. Furthermore, the 'western' and 'healthy' dietary patterns among boys and the 'traditional and western' pattern among girls were correlated to socioeconomic status.

The identification of dietary patterns in a representative sample of the adolescent population and their relation to socioeconomic status, nutritional behaviour and nutrient intake has been rarely examined. Empirical evaluated dietary patterns are specific for the examined study population and reflect culturally influenced eating habits. Furthermore, studies used different methods e. g. for food grouping. Therefore, deviations between dietary pattern compositions are obvious. Nevertheless, the patterns found among German adolescents were, to some extent, similar to those found in previous studies in this age group in other countries. Comparison of dietary patterns among adolescents between different countries can give useful information on similarities in food consumption behaviour between different populations. For such comparison however cultural and economic conditions should be similar so that it is likely that the food supply is similar. If similar dietary patterns are found in such countries, it could be interesting to obtain further information concerning the associations between patterns and health in this age group.

Our 'western' pattern was partly comparable to the 'western' patterns found among adolescents in other countries e. g. in the Western Australian Pregnancy Cohort Study (Raine Study) [40] and in the Korean Nutrition Health and Nutrition Examination Survey (KNHANES) [29] and also to the 'high fat and sugar' pattern found in a representative sample of adolescents in the 1995 Australian National Nutrition Survey [27]. All these patterns were characterized by pizza, hamburger, soft drinks and meat or meat products. In contradiction to our study, alcohol consumption was not analysed in those studies. In Germany, we observed that adolescents already consume relevant amounts of alcoholic drinks [41].

Our 'healthy' patterns were to some extent comparable to other 'healthy' patterns found among adolescents e. g. in a population based sample of Spanish adolescents, and in samples of adolescents in Finland (Cardiovascular Risk in Young Finns Study), Japan (Japanese female dietetic course students), and Australia (Raine Study) [4,18,23,40] and to the 'fruit, salad, cereals, and fish pattern' found in the 1995 Australian National Nutrition Survey [27].

Our findings on the association between dietary pattern and nutrient intake were especially similar to results of the 1995 Australian National Nutrition Survey [27]. Adolescents in Australia with a higher score of the 'fruit, salad, cereals, and fish' pattern had a higher energy-adjusted intake of dietary fibre, beta carotene, folate, vitamin C and protein similar as German adolescents with higher scores of the 'healthy' patterns. The 'high fat and sugar' pattern was associated with a lower energy adjusted intake of dietary fibre, folate and iron [27].

Among German adolescents, despite the high intakes of meat in the 'western', 'traditional' and 'traditional and western' patterns, iron density decreased with increasing scores of these patterns, whereas iron density increased with increasing scores of the 'healthy' patterns. This can be explained by the fact that meat and processed meats are less important sources of iron than bread, juices, vegetables, fruits and breakfast cereals among German adolescents [38]. However, this does not consider differences in bioavailability of iron between animal and vegetarian foods. Furthermore, white bread, a characteristic component of the 'traditional' pattern, has lower iron content than whole grain bread.

Most former studies [23,27,40] determined dietary patterns for boys and girls together. Among German adolescents, we found different dietary patterns between boys and girls. Even between the 'healthy' dietary patterns, differences concerning the food group loadings are notable. Further differences between boys and girls were found. Firstly, for boys the most predominant dietary pattern was the 'western' pattern, among girls the 'healthy'. Second, among boys many associations between the 'healthy' dietary pattern and socioeconomic or behavioural factors (e. g. physical activity, frequency of joined family dinner) were found. Among girls there was no association between socioeconomic or behavioural factors and the 'healthy' dietary pattern. Thirdly, the two predominant dietary patterns found among German girls explained only 13.2% of variance in food group intake, while among German boys, the three predominant dietary patterns explained 18.1% of variance. This may indicate that there is more individual diversity within the diet of girls or that boys have more often similar food preferences. Fourthly, particularly notable among German boys was the increasing age from the lowest to the highest quartile of the 'western' pattern. Among girls, with increasing scores of the 'traditional and western' pattern age was decreasing. The present study used a cross sectional design. Thus, we are not able to evaluate long term changes. But it may be assumed, that boys change their eating behaviours during adolescence toward a less favourable dietary pattern, whereas girls do not.

Among U.S. middle and high school students in Minnesota (Project EAT) [14] and students in Vyronas region, Athens [21] high scores of 'take away food' or 'fast food' patterns were associated with increasing age among both sexes.

Previous studies investigated the relationship between dietary patterns and socioeconomic parameters. In a representative sample of adolescents in Australia and among Greece students, no association was found [21,27]. In a German study, there was no association between clusters of fat intake patterns and mother's educational background [42]. In Australia (Raine Study), Finland (Cardiovascular Risk in Young Finns Study), and Scotland (The Survey of Sugar Intake among Children in Scotland), the 'healthy' or 'vegetables' patterns were associated with higher education of the mother [40], both parents [4] or the parent who mainly provides the food [25]. The 'western' pattern in the Australian Rain study and the 'traditional' pattern in Finland (Cardiovascular Risk in Young Finns Study) were associated with lower educational background. Furthermore, in Scotland (The Survey of Sugar Intake among Children in Scotland), the 'vegetables' pattern was associated with higher socioeconomic status and higher household income [25] and among adolescents of the Balearic Islands higher scores of the Mediterranean pattern were associated with higher parental socio-economic status among girls [24].

With increasing 'healthy' pattern scores, the percentages of boys taking dietary supplements increased. Among girls, this increase was not significant. Previous studies in Japan (Japanese female dietetic course students) [43] and Finland (Cardiovascular Risk in Young Finns Study) [4] found an increasing percentage of supplement users with increasing scores of healthy dietary patterns among both sexes.

In Finland (Cardiovascular Risk in Young Finns Study) and Japan (Japanese female dietetic course students), 'traditional' dietary patterns were found more often in rural areas [4,18], whereas a 'healthy' pattern was observed more often in urban areas [4], which follows our results. Dietary patterns are very likely influenced by local food supply. In rural areas of Germany there are less fast food and non-traditional restaurants compared to urban areas. Maybe therefore, boys living in rural areas more often follow a traditional German diet.

Other studies have described an association between a higher level of physical activity and higher adherence to healthier dietary patterns [21,23,44], like among boys in our study. Conversely, with increasing hours of watching television, a higher adherence to less healthy dietary patterns (e. g. 'snacky' or 'western'), similar to the association found among German girls with higher scores of the 'traditional and western' pattern in our study, was also previously observed [21,24,25,40].

We also used energy-adjusted dietary pattern scores [45] (data not presented). This did not essentially change the results concerning the association between dietary pattern scores and socioeconomic and lifestyle characteristics.

In our study, no association between dietary pattern scores and overweight was found. Only few studies have previously investigated the association between dietary patterns and BMI among adolescents. These studies came to different results. In a German study, BMI showed no association to fat intake patterns within a longitudinal analysis [42]. A study in Scotland also found no association between dietary patterns and BMI [25]. Another study observed a lower BMI among those with a healthier dietary pattern and a higher BMI among those with a western dietary pattern [43]. One study even found a higher BMI among persons with a healthier nutrition [46].

One reason for the different results may be that overweight persons who attempt to lose weight tend to confound the relationship between nutrition and BMI [46]. Furthermore, obese people tend to underreport fatty foods and foods rich in carbohydrates [47]. Besides, it is difficult to detect minor overconsumption of energy in epidemiological studies that track the development of obesity over long periods [42] and overweight is primarily controlled by energy balance. Another source of error may be the bias of self-reported body weight and height, which often differ from measured figures [48]. In a subsample of KiGGS, validity of self-reported height and weight among 11- to 17-year-old adolescents was assessed. BMI values calculated from self-reported weight and height were lower than those calculated from measured values among both sexes [49]. Thus, we probably underestimate BMI in our study. However, standardized anthropometric measurements were not feasible due to the nature of household visits and interviews taken place in study vans.

Further analysis concerning dietary patterns and BMI were conducted while excluding low energy reporters (about 10% of girls and 7% of boys) [50]. This did not change the results essentially.

Strengths of this study include the utilization of representative population-based data and the comprehensive nutrition interview, which documented food consumption during a period of four weeks, as well as the inclusion of data on nutrition behaviour, lifestyle habits and socioeconomic background.

A limitation of our study is the cross-sectional design, which allows no statements concerning causation.

Potential limitations of the interview-based dietary assessment methods in general are underreporting of usual intake and invalid reporting due to memory gaps and social desirability. DISHES was only validated for adults but in a recent study among adolescents aged 12 to 17 years it showed fair to moderate ranking validity with food group intake assessed with a FFQ [51]. In addition, a pretest on feasibility of the use of DISHES in the age group of 12 to 17 years was conducted, which indicated no particular problems for conducting DISHES in this age group. Furthermore, most interviews where carried out at home of the participants, which gave the opportunity to get additional information from the parents (e. g. concerning name of meals, kind of meat).

Another limitation of the study was the self-reported physical activity which may be affected by misreporting, e. g. overreporting influenced by social desirability [52]. However, within the setting of this survey, more objective methods to determine physical activity would be too time-consuming and expensive and where therefore not feasible.

For children and adolescents with a migration background, a higher proportion of unreachable addresses and non-respondents were expected. Thus an oversampling of this population group was performed in KiGGS. Furthermore, invitation letters, information material and questionnaires were translated into six languages [31]. However, for the dietary assessment in EsKiMo, certain basic German language skills were required. Thus, the proportion of participants with a migration background in EsKiMo was somewhat lower than in KiGGS. However, 6.8% of the EsKiMo participants had a one-side and 11.3% a two-side migration background (unweighted percent). Nevertheless, separate analysis concerning migration background was not reasonable in EsKiMo because of a relatively small sample size which included only 65 boys and 79 girls with a migration background which came from several countries.

Principal component analysis is generally exposed to the limitation of some subjectivity, particularly when grouping the food items, selecting the method of factor rotation, defining the number of patterns to be retained and labelling of the factors [53]. To enable comparability with other studies, we used criteria similar to those reported by other dietary pattern analyses [14,21]. In addition, we tested a further version of food grouping with 32 food groups instead of 48. This led to very similar results concerning the number of principal components to be retained and the food groups with high loadings within patterns. Correlation coefficients between pattern scores determined with 48 food groups and those determined with 32 food groups ranged between 0.93 and 0.97. McCann also noted that food classification method affected neither the number nor character of the identified patterns [54].

Dietary patterns derived using principal component analysis generally tend to account for only a small amount of the total variance of diet [55]. The variance explained in the present study was similar to a study among 12 to 17 year-old Scottish adolescents, in which 3 dietary patterns based on 141 food groups accounted for 14.4% of variance among boys and 15.1% among girls [25]. A slightly higher variance than in our study was observed among 12 to 18 year-old Australian adolescents, in which 3 dietary patterns based on 86 food groups accounted for 21.7% of the variance in the data [27].

## Conclusions

In conclusion, we detected three distinct dietary patterns among German boys and two among German girls. Dietary patterns were associated with differences in nutrient intake, socioeconomic status and lifestyle characteristics. Higher scores for unhealthier dietary patterns (with higher consumption of take away foods, meat, confectionary and soft drinks, higher energy density, higher percent of energy from unsaturated fatty acids, lower percent of energy from carbohydrates and lower nutrient densities of several vitamins and minerals) are observed more often among 16- to 17-years old boys and adolescents with lower SES. Therefore, nutritional interventions should try to focus more on adolescents with lower SES and boys in general.

## Abbreviations

SES: Socioeconomic status; PA: Physical activity; SFA: Saturated fatty acids; PUFA: Polyunsaturated fatty acids.

## Competing interests

The authors declare that they have no competing interests.

## Authors' contributions

GBMM designed and managed EsKiMo; AR managed the field work of EsKiMo; GBMM, ST, JR, MBS, AR designed and wrote the study proposal for the pattern analysis; AR conducted the presented analysis and drafted the manuscript; CH and MBS assisted with statistical analysis and interpretation of the results; GBMM, JR, ST contributed to the interpretation of the results and the writing of the manuscript. All the authors were involved in the critical revision of the manuscript for important intellectual content.

## Pre-publication history

The pre-publication history for this paper can be accessed here:

http://www.biomedcentral.com/1471-2431/12/35/prepub
